# Re-Defining the Population-Specific Cut-Off Mark for Vitamin A Deficiency in Pre-School Children of Malawi

**DOI:** 10.3390/nu13030849

**Published:** 2021-03-05

**Authors:** Blessings H. Likoswe, Edward J. M. Joy, Fanny Sandalinas, Suzanne Filteau, Kenneth Maleta, John C. Phuka

**Affiliations:** 1 Department of Public Health, School of Public Health and Family Medicine, College of Medicine, University of Malawi, Private Bag 360, Chichiri, Blantyre 3, Malawi; kmaleta@medcol.mw; 2 Faculty of Epidemiology and Population Health, London School of Hygiene & Tropical Medicine, Keppel Street, London WC1E 7HT, UK; edward.joy@lshtm.ac.uk (E.J.M.J.); fanny.sandalinas@lshtm.ac.uk (F.S.); suzanne.filteau@lshtm.ac.uk (S.F.)

**Keywords:** vitamin A, retinol binding protein, serum retinol, inflammation, c-reactive protein, alpha-1 acid glycoprotein

## Abstract

Retinol Binding Protein (RBP) is responsible for the transport of serum retinol (SR) to target tissue in the body. Since RBP is relatively easy and cheap to measure, it is widely used in national Micronutrient Surveys (MNS) as a proxy for SR to determine vitamin A status. By regressing RBP concentration against SR concentration measured in a subset of the survey population, one can define a population-specific threshold concentration of RBP that indicates vitamin A deficiency (VAD). However, the relationship between RBP and SR concentrations is affected by various factors including inflammation. This study, therefore, aimed to re-define the population-specific cut-off for VAD by examining the influence of inflammation on RBP and SR, among pre-school children (PSC) from the 2015–16 Malawi MNS. The initial association between RBP and SR concentrations was poor, and this remained the case despite applying various methods to correct for inflammation. The World Health Organization (WHO) recommends the threshold of 0.7 µmol/L to define VAD for SR concentrations. Applying this threshold to the RBP concentrations gave a VAD prevalence of 24%, which reduced to 10% after inflammation adjustments following methods developed by the Biomarkers Reflecting Inflammation and Nutritional Determinants of Anemia (BRINDA). Further research is required to identify why SR and RBP were poorly associated in this population. Future MNS will need to account for the effect of inflammation on RBP to measure the prevalence of VAD in Malawi.

## 1. Introduction

Vitamin A deficiency (VAD) is a global challenge, affecting about 48% of pre-school children (PSC) in Sub-Saharan Africa [1]. This deficiency can cause problems with vision, cell differentiation to maintain the epithelial system, and immune response [2,3]. It is assessed using serum retinol (SR) as recommended by the World Health Organization (WHO) [4], but it can also be assessed using retinol-binding protein (RBP), the carrier protein for SR [5]. RBP is preferred as a surrogate for SR in large population surveys because it is cheaper to analyse, and its sample management is simpler [6].

The WHO recommends an SR cut-off of 0.7 µmol/L to determine VAD [7], but there is no agreed cut-off value for RBP. The relationship between SR and RBP is not always equimolar and recent publications have shown that there is a markedly higher concentration of RBP than SR in the blood pool which gives a molar ratio of ~0.9 [5]. This occurs because of the presence of Apo-RBP in the blood (RBP which is not complexed with SR) [3]. As such the molar cut-off for RBP may need to be higher than that of SR to accurately define VAD. Linear regression of RBP and SR has been suggested as an approach to identify a population-specific cut-off for RBP which is equivalent to the SR cut-off of 0.7 µmol/L for defining VAD [8]. National-level micronutrient surveys are increasingly adopting this method for use to define population-specific cut-offs [9,10]. However, the relationship between these two markers can be affected by some factors such as inflammation which affects performance of the linear regression.

Acute infection and inflammation processes in the body temporarily affect concentrations of some serum and plasma micronutrients [11]. Inflammation can be characterised by increased levels of C-reactive protein (CRP), and Alpha-1-acid glycoprotein (AGP) which are inflammatory markers. During an acute phase response, the concentration of some micronutrients increases in the blood whereas others decrease to control the damage that is caused by the infections [12]. Both RBP and SR are negative acute-phase reactants (APRs) [13,14] because their concentrations in the blood pool decrease temporarily during inflammation [15,16,17,18]. However, changes in concentration of these two markers do not necessarily occur at the same pace [11]. A study of acute inflammation in PSC demonstrated that SR is the first marker to be affected by inflammation and its concentration decreases at a faster rate and to a much lower level than that of RBP [19]. These differences in the rate of depression can affect the molar ratio of SR and RBP [3]. Methods have been developed to adjust the concentrations of APR biomarkers to account for the effects of inflammation, to better reflect the subject’s nutritional status [13,18,20,21].

In the 2015–2016 Malawi Micronutrient Survey (MNS) linear regression indicated a weak linear relationship between SR and RBP (r = ~0.20) [22,23]. Furthermore, a high prevalence of inflammation (>50%) was reported in PSC, which may have affected the relationship between RBP and SR. The linear regression approach gave a cut-off RBP concentration of 0.46 µmol/L; in contrast, most studies have defined cut-offs in the range of 0.60–1.29 µmol/L [6,8,24,25,26,27,28]. This study, therefore, aimed to re-define the population-specific cut-off for VAD by accounting for effects of inflammation on the linear relationship between RBP and SR, in a sub-sample among PSC 2015–2016 Malawi MNS. The specific objectives were to (a) adjust for confounders of inflammation when applying the linear regression method, (b) re-determine the prevalence of VAD in pre-school PSC using the updated population-specific cut-off.

## 2. Materials and Methods

The analysis used secondary data from the recent Malawi MNS which was conducted in 2015–16. The MNS was a sub-study of the Demographic Health Survey (DHS), where both studies aimed to give a cross-sectional view of the health and nutritional status of the country. The study design is reported in the Malawi MNS report [23] and presented briefly here.

Participating households were selected using two-stage cluster sampling in all 28 districts, across the three regions of the country. The sample size was based on predicted changes in the prevalence of VAD in PSC between the 2009 and 2015–2016 MNS (22% to 16%). The calculation was based on a confidence interval of 95%, power of 80%, design effect of 2.0 and predicted 90% response rate from each household. A sample size of 1452 PSC was deemed adequate for estimating the national and region-specific prevalence of key nutrition factors at 5% and 10% precision, respectively. A total of 2277 households were selected for the MNS, and among these, 2250 were deemed eligible for inclusion in the study. There was a 94% participation rate which resulted in 2114 households participating from which all PSC (n = 1233) were recruited.

The vitamin A status of the MNS population was assessed using RBP concentrations, with a population-specific threshold, to define VAD derived by regressing RBP against SR concentrations, measured in a random subsample of PSC. The first household in each cluster was randomly selected and approached first for this subset participation. Venous blood samples of 7 mL were collected from participants into trace element free vacutainers. These samples were spun to extract serum of which 100 µL was aliquot into a PCR vial which was shipped to the VitMin lab (Willstaett, Germany) for RBP analysis by sandwich ELISA [29]. The ELISA method also analysed two inflammatory markers: CRP and AGP which were reported alongside RBP concentrations.

Another blood sample was collected from the subsample group for modified relative dose response (MRDR) and SR analysis. For MRDR, a small dose of SR analogue 3,4 didehydro SR (DR) was administered together with a fatty snack to the participants and blood samples were collected 4–6 h later [30]. A serum aliquot of 250 µL was removed from this sample into a sterile vial for SR and SR analogue analysis. These samples were analysed by the INCAP lab (Guatemala city, Guatemala), using High-Performance Liquid Chromatography (HPLC). Assay quality control (QC) data denoting accuracy and precision were assessed using standard methods for RBP, CRP and AGP and found to be acceptable, while assay QC data was unavailable for retinol (see Discussion).

### 2.1. Statistical Analyses Used

All SR concentrations above 3 µmol/L were considered as outliers and were removed from this analysis. Data for both SR and RBP concentrations were examined for normality using histograms and measures of central tendency. Preliminary analyses were done to test if there were effects of inflammation on SR or RBP data. Multivariate (both CRP and AGP included) and bivariate (CRP and AGP added separately) analyses were run to test for associations with SR and RBP separately.

Several steps were followed to attempt to improve the relationship between SR and RBP. Firstly, the original analysis from the MNS survey was replicated and the Pearson’s correlation coefficient between SR and RBP concentrations was calculated. Then, a linear regression was conducted between SR and RBP giving the equation:

SR=α+β(RBP)          Model 1

which was used to determine the RBP value that corresponded to the SR value of 0.7 µmol/L.

Approaches to account for inflammation were applied to attempt to improve the relationship between SR and RBP. Three methods were used to account for inflammation as a primary confounder. The first method was the internal correction factor (ICF) criteria originally proposed by Thurnham et al. [31]. For this, a new variable was created to define inflammation status in four stages: normal (CRP < 5 mg/L and AGP < 1.0 g/L), incubation (CRP > 5 mg/L and AGP < 1.0 g/L), early convalescence (CRP > 5 mg/L and AGP > 1.0 g/L), and late convalescence (CRP < 5 mg/L and AGP > 1.0 g/L). This variable was then added to the linear regression to give:

SR~RBP+INFLAMMATION STATUS   Model 2.

The second method involved adding CRP and AGP as continuous variables to the model, without defining inflammation criteria, resulting in the model:

SR~RBP+CRP+AGP          Model 3

The third method involved removing from the dataset participants with inflammation defined as CRP > 5 mg/L and/or AGP > 1 g/L before running the linear regression. This equation was similar to model 1 but had a lower sample size due to the removal of inflamed participants. This gave the model:

SR=α+β(RBP)          Model 4

Results obtained from models 2 to 4 were compared to the original unadjusted regression (model 1) to determine if there was a cut-off derived, that was more consistent with literature. All outcomes were interpreted with reference to the three statements below:

- A strong correlation between the two variables, preferably r ≥ 0.7 [32].
- A clear linear relationship between the SR and RBP observed in the scatter plot.
- A linear regression equation that gives a cut-off value which reflects evidence that the SR: RBP ratio is < 1.0, especially in the presence of inflammation.

The newly determined cut-off was then applied to the inflammation-adjusted RBP data to determine a new prevalence estimate of VAD. The data was adjusted for inflammation using the BRINDA method. This method uses log_e_-transformed values and adjusts values for all participants whose CRP and AGP values are above the maximum 10th decile. This adjustment method is explained in detail elsewhere [33].

All analyses of this study were made using R programming language, version 3.6.1 [34], in RStudio [35]. Results are presented as means ± SD and point estimates are presented with the 95% confidence interval (CI). Two-tailed *t*-tests for un-paired data were applied to test for significant differences of means between the sub-sample and full sample groups. For point estimates, two-tailed proportion z-tests or Fisher’s exact probability test (based on sample size) were used. A *p*-value of 0.05 was considered statistically significant. All results were weighted to account for survey design. Each household was weighted during sample collection to account for the stratified sampling. In this study, the weights for each participant (based on the household of origin) are applied using mathematical calculations that are recommended by the Demographic Health Survey (DHS) to give indicators at a national level.

### 2.2. Ethical Considerations

The datasets used in the current analysis were downloaded with permission from the DHS website (http://dhsprogram.com, accessed on 15 January 2020). The MNS study as a sub-study of DHS was approved by the National Health Sciences Research Committee of Malawi, reference number NHSRC 15/5/1436. Procedures followed for informed consent and assent are reported in detail in the MNS report [23]. Ethical approval for the re-analysis of the VAD data for this study was obtained through the London School of Hygiene & Tropical Medicine Observational Research Ethics Committee (Reference 21903, 17 April 2020).

## 3. Results

**Figure 1:**
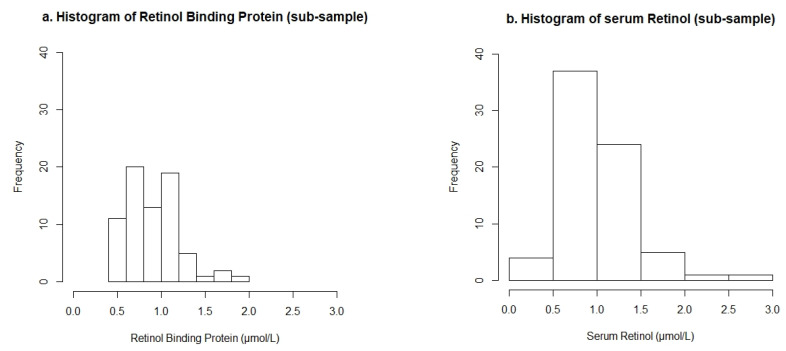
Histograms of (**a**) Retinol Binding Protein and (**b**) Serum Retinol concentrations from the sub-sample of n = 72 preschool children.

A total of 1233 PSC were surveyed in the MNS survey with similar numbers of male and females. Of these, 1102 participants agreed to have venipuncture blood collected; the other 131 PSC with no available biological data were excluded from this analysis. The regression analysis only focused on a sub-sample (n = 74) for which SR was analysed. Two observations for SR were considered outliers using the pre-defined cut-off values and were removed from the analysis, leaving 72 participants with RBP and SR data (sub-sample) and 1100 participants with RBP data only (full sample). A flow chart detailing participant inclusion is found as supplementary Figure S1. Histograms showing the distribution of RBP and SR concentrations in the sub-samples are presented in Figure 1. The distribution of RBP concentrations was skewed towards the low end of the range, with 24% of the observations being <0.7 µmol/L. Data points for SR followed a bell-shaped distribution and 79% of the data points were >0.7 µmol/L. The mean concentration of SR was greater than that of RBP.

### 3.1. Descriptive Results

The mean concentration of RBP in the subsample was 0.90 ± 0.25 µmol/L and was similar to the full sample set (0.89 ± 027 µmol/L). The mean concentration of SR in the sub-sample was 0.98 ± 0.35 µmol/L as shown in Table 1. For CRP and AGP, the geometric means were used to account for skewed data. These are presented in Table 1 after exponentiation. The geometric mean of CRP in the sub-sample was 1.00 mg/L with a median of 0.66 mg/L and an interquartile range (IQR) of 0.26–3.22 mg/L, respectively, whereas for AGP the geometric mean was 0.95 g/L, the median 0.84 g/L and the IQR of 0.59–1.43 g/L. In the full sample, the geometric mean of CRP was 1.47 mg/L whereas for AGP it was 1.15 g/L. The medians and IQR for the full sample were 1.39 mg/L (0.41–4.70 mg/L) and 1.11 g/L (0.74–1.83 mg/L) for CRP and AGP, respectively. There was a high prevalence of inflammation in the sub-sample with 43% [95% CI: 28; 59] of the PSC having elevated levels of inflammatory markers. Most of those inflamed were in the late stage of inflammation (21%) followed by the early stage of inflammation (18%).

### 3.2. Associations between Vitamin A Biomarkers and Inflammatory Biomarkers

Bivariate and multivariate analyses indicated weak associations between both vitamin-A biomarkers and inflammatory markers. For the bivariate analysis, CRP and AGP were negatively associated with both RBP and SR (Table 2). Differences between bivariate and the multivariate analyses were minimal for both RBP and SR.

### 3.3. Linear Regression Outputs

**Figure 2:**
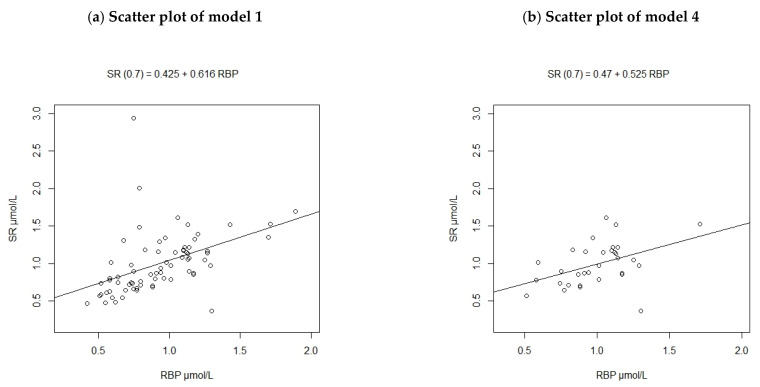
Scatterplots of Retinol Binding Protein against Serum Retinol concentrations from the sub-sample of pre-school children (**a**) before removing inflamed participants (n = 72), (**b**) after removing inflamed participants (n = 32).

The scatter plot for model 4 was produced and compared with that of model 1, due to differing sample sizes (Figure 2). When PSC with inflammation (defined by raised CRP > 5 mg/L and/or AGP > 1 g/L) were removed from the sample size (model 4), changes were observed for SR data but not for RBP data. The maximum SR value in the whole sub-sample (n = 72) was 2.94 µmol/L and this decreased to 1.68 µmol/L for model 4 (n = 32) when inflamed participants were removed. The mean concentration of SR in the whole sub-sample was 0.98 ± 0.35 µmol/L and there was not much change (1.00 ± 0.23 µmol/L) after the inflamed individuals were removed. The data points for model 4 were more concentrated on the lower range of both RBP and SR concentrations, compared to those of model 1.

Table 3 presents the regression outputs and calculated cut-off values. Model 1 showed a poor correlation between SR and RBP, with a correlation coefficient of determination of 0.2. The model also indicated that a 1 µmol/L increase in RBP was associated with a 0.62 µmol/L increase in SR. For this model, an RBP cut-off of 0.45 µmol/L was equivalent to 0.7 µmol/L of SR. Adjusting for different stages of inflammation (model 2) showed that participants who were in the early and late convalescence stages had lower SR concentrations than those in incubation. This model was a poorer fit than the original model because it gave an even lower correlation coefficient of SR and RBP. When CRP and AGP were added to the regression model directly (model 3), the correlation estimate was slightly higher, however, the obtained cut-off value was still low (0.43 µmol/L).

When PSC with inflammation were removed from the analysis, SR concentration was on average 0.37 µmol/L higher than RBP and the derived threshold was 0.43 µmol/L. The relationship between SR and RBP was the lowest (across all methods) with this method, as only 12% of the variation in SR could be explained by RBP. The adjusted model did not give cut-off values which were higher than the original one.

### 3.4. Prevalence of Vitamin A Deficiency

After reviewing all the regression outputs presented in Table 3 against the pre-determined criteria, it was determined that regardless of the confounder adjustments, SR and RBP were still not better correlated and one marker could not be used to predict the other. Therefore, the WHO cut-off of 0.7 µmol/L was used to determine VAD. For the BRINDA adjustments, both CRP and AGP were negatively associated with RBP in the full dataset and 57% of PSC had elevated levels of one or both acute-phase proteins. Most of these inflamed participants were in the early and late stages of inflammation. The BRINDA method adjusts for the top 90% of elevated acute-phase proteins [36]; as such 920 participants were adjusted for inflammation and 180 were unadjusted. The adjustment increased the mean RBP concentration from 0.89 ± 0.27 µmol/L to 1.01 ± 0.29 µmol/L.

When the cut-off of 0.7 µmol/L was applied to the RBP data, before adjusting for inflammation, the prevalence of VAD was 24% (Table 4). This reduced to 10% following adjustment by BRINDA. When the original cut-off mark of 0.43 µmol/L was used, the estimated prevalence was 2% before adjusting for inflammation, and 0% afterwards.

## 4. Discussion

### 4.1. Adjustment for Inflammation

The relationship between SR and RBP was tested in a subsample of n = 72 participants, to determine the cut-off mark for VAD using RBP data in the recent MNS in Malawi. The results showed a poor linear relationship between these two biomarkers which in turn gave a cut-off for RBP concentration lower than what has been published previously [6,8,24,25,26,27]. Three different methods were applied to correct for inflammatory effects, in an attempt to improve the relationship between RBP and SR, but none of these methods improved the performance of the regression model.

Hence, the poor performance of the regression cannot be explained by the effects of inflammation, and this is further explored in the next section. Nonetheless, it is important to consider inflammation when interpreting SR and RBP concentration data. During the acute phase response, SR decreases further and at a faster rate than RBP. As such, lower concentrations of SR than RBP would be expected in the presence of acute infection. This changes the molar ratio of these two markers and the gap in acute phase reduction cannot be easily adjusted for. Other studies have found that although SR is associated with inflammation stage during infection, it is not associated with time [37]. Therefore, the rate at which SR decreases is primarily determined by the stage of inflammation, i.e., whether a person is in early, incubation or late stage of inflammation. Additionally, if an individual had a severe infection, their SR concentration would decrease further in a short time than an individual with a mild infection over the same time period. The effects of inflammation on RBP and SR are complex such that the current methods of inflammation adjustment (which are applied in this study) are imperfect and there may be a systematic bias in the estimation of VAD.

Two previous studies have established that the effects of inflammation on SR are mostly observed when CRP levels are elevated (early and incubation stages) but not when CRP levels are depressed (late stages) even if AGP is high [19,37]. For the current study, similar results were also observed when associations between SR and the inflammatory markers were explored. In the combined analysis, AGP had a positive association with SR whilst CRP had a negative association with SR. This could have resulted because of collinearity as the bivariate analyses showed negative association for both inflammatory markers. Other studies have also reported similar findings [31,38], and this has a consequence for the inflammation adjustment methods that are used. It would appear that to accurately adjust for SR, only CRP should be accounted for as opposed to both CRP and AGP. Since SR supposedly normalises by late convalescence (when AGP is still elevated), adjusting the participants in this stage for inflammation could lead to over adjusting. On the other hand, RBP is associated with both CRP and AGP and as such would need to be corrected for using both inflammatory markers [18,39]. This introduces a lot of complexities when the relationship between these two markers is considered. Linear regression may not be sufficiently robust to account for all these factors and to determine the appropriate cut-off mark for VAD in RBP data. There is a need for a more integrated method that not only applies standard inflammation adjustment methods but also takes into account the various inflammation effects on SR and RBP. The relationship between RBP and SR is complex and further research is required to explore the effects of various confounders.

### 4.2. Analytical Data

Comparison of the SR and RBP concentrations in the sub-sample of PSC found higher concentrations of SR than RBP. An SR: RBP molar ratio > 1 is contrary to what literature suggests is biologically plausible, especially if the effects of inflammation and the presence of Apo-RBP are considered [3,24,40]. In studies where the cut-off mark as determined by linear regression was deemed accurate, the regression equation indicated a higher concentration of RBP than SR, or an almost equivalent relationship [41,42,43]. This current study showed that SR values were on average 0.3 µmol/L higher than RBP values as shown in Table 3.

Previously, analytical issues have been cited as a causative factor for lack of agreement between RBP and SR molar ratios in MNSs [44]. A systematic bias introduced during sample analysis could lead to an implausible SR: RBP ratio. This could explain the higher SR values that were observed and failure of the inflammation adjustments to improve the relationship between SR and RBP. To further investigate this, we requested analytical quality control (QC) data from the relevant laboratories.

There is limited availability of Certified Reference Materials in this analytical field, but a standard reference material (serum, BIORAD) at three concentrations was used as an independent check of RBP, CRP and AGP analyses by the VitMin laboratory. These were introduced at the analytical laboratory; they were prepared in the same way as the MNS samples and run with each batch. For RBP, the low, medium and high concentration references had inter-assay coefficients of variation (CV) of 5.73%, 5.39% and 11.81%, respectively. For CRP, the CV were 7.36%, 6.04% and 4.21%, for low, medium and high references, respectively. For AGP the CV for the low, medium and high concentration of reference materials were 13.67%, 11.76%, and 12.83%, respectively. The limit of detection for RBP, CRP and AGP was 0.1 µmol/L, 0.2 mg/L and 0.1 g/L, respectively. The average inter-assay CV across the three concentrations of reference materials for RBP, CRP and AGP was 7.65%, 5.87% and 12.75%, respectively. There was no evidence of any systematic bias for these analytes. No standard reference materials were run in the analysis of SR concentrations at the INCAP laboratory, so it is not possible to independently benchmark the quality of those analytical data.

Good sample management, assay selection and assay performance are crucial to ensure accurate results. Unfortunately, there is inconsistent reporting practices for MNS data [45], which poses a great challenge of understanding data quality. If reporting of QC data were standardised for surveys, it would improve the recognition of the quality of work done by the laboratories and increase the confidence in comparing results between surveys in different locations, or through time. The sample management of SR is more challenging to handle because the SR is photo- and temperature-sensitive as opposed to RBP which is more stable [6,8]. Furthermore, SR requires more complex assays to analyse, e.g., HPLC [46], whereas RBP can be analysed by simplified methods, such as Enzyme-Linked Immunosorbent Assay (ELISA) [29]. If proper laboratory practices are not followed, it is easier to have assay complications arising from the complexity of HPLC, leading to inaccurate results. Whilst the RBP and inflammation QC data indicate that survey sample data for these parameters were comparable with each other, it was not possible to assess this for the SR data. In future surveys, it is suggested that the inclusion of an independent reference material would be beneficial to better understand the specific population relationship between SR and RBP, as well as inflammation biomarkers.

There are other factors as well that affect the relationship between SR and RBP such as zinc deficiency [20,47], and severe acute malnutrition (SAM) [24,48,49] which are common in PSC of Malawi [23]. Low zinc levels and SAM lower the concentration of holo-RBP in the blood, which further decreases the molar ratio of SR to RBP [50,51]. There are no validated methods for correcting for these confounders when linear regression (or any other method) is used. Nonetheless, with the reported high prevalence of zinc deficiency in this population group [20], a high molar ratio of SR to RBP (as found in the results) is inconsistent. The findings of the current study, not only point to possible data quality issues but also highlight the limitations of the linear regression approach to determine the threshold for VAD using RBP concentrations. Studies that explored the linear regression relationship and found thresholds that were agreeable, have had greater sub-sample sizes than the Malawi MNS. The sub-sample sizes ranged from 123 to 239 individuals, representing 15 to 26% of the total sample [8,24]. There is thus a need to statistically determine the size of the sub-sample to support the regression approach, to ensure that it is large enough to provide a robust estimate of the relationship between the two markers, and in some cases it may be preferable to measure SR in the whole population.

In the Malawi MNS, MRDR was assessed as well to determine the concentration of vitamin A in the subsample in which SR concentration was determined [23]. Results of MRDR indicated a low prevalence of VAD (MRDR ≥ 0.060), however, MRDR results are a ratio of DR to SR, which means the MRDR results will be affected by any problems with SR values. Furthermore, the MRDR samples for this survey were collected, transported and analysed together with the SR, as such any issues with sample management throughout the analysis chain, e.g., contamination or use of different calibrators [44], that might have affected SR would likely have affected MRDR too.

### 4.3. Alternative Approaches to Determining VAD Prevalence

Assessment of VAD using RBP concentrations will remain a challenge until an appropriate deficiency threshold can be determined. Literature exists to support the optimization of cut-off markers of a single biomarker, using validated statistical approaches [52,53,54]. The minimum *p*-value approach, for instance, is a method that selects the cut-off value which gives the most significant statistical difference between the two diagnostic groups [55]. In the present case, such methods could be used as alternatives for determining the cut-off of RBP concentration without using SR concentration as a predictor. This method could be applied on existing single, or pooled datasets of micronutrient surveys to test for optimal cut-off points for RBP. The findings could then be incorporated in the design of randomized controlled trials for a more robust approach and to validate the newly identified cut-off marks [54], because cross-sectional survey data cannot be used to validate cut-offs [56].

Linear regression could not be used to determine the population-specific cut-off of PSC because of the poor performance of the method. None of the confounder adjustments were able to improve the relationship between SR and RBP to a satisfactory level. As such, the threshold concentration of SR of 0.7 µmol/L suggested by WHO was used to determine VAD in the RBP concentrations [7]. This value assumes a 1:1 molar ratio between the two biomarkers but with all biological factors in play, the possibility of this ratio occurring is low. However, it may be a preferable approach compared to that of a regression method with a poor correlation.

Similar approaches have been followed by MNS conducted in Sierra Leone [10], and Ghana [9], where linear regression gave a cut-off mark that was below the expected range. Notably, for these surveys, there was a good correlation between SR and RBP, but still, the linear equation gave a lower than expected cut-off mark. Nonetheless, issues with linear regression were noted by the responsible researchers of those surveys done in Sierra Leone and Ghana and prompted them to make an informed decision to use the WHO cut-off for SR to define VAD in RBP concentrations. The implications of a poorly defined threshold for VAD in MNS are serious, since the results are used by multiple stakeholders to make decisions on intervention programmes (e.g., fortification or supplementation) that affect millions of lives.

For other surveys like that done in The Gambia, there was a strong correlation between SR and RBP concentrations (R^2^ = 0.9), and the linear regression equation gave a cut-off mark of 0.7 µmol/L, which is within the acceptable range and was used to determine VAD prevalence [42]. Similarly, in Oman, SR and RBP concentrations correlated well (R^2^ = 0.9) and gave a cut-off mark of 0.73 µmol/L, which was used to determine VAD prevalence [43]. For this survey, only 20.2% of the PSC had elevated levels of CRP and AGP, whereas, for the Gambia, this was not clearly described. Based on this, it appears linear regression does work favourably in other situations, possibly when there are lower levels of inflammation among the participants. However, there is no apparent consensus on the use of, and interpretation of linear regression for VAD cut-off in national MNS.

The SR cut-off of 0.7 µmol/L that is recommended by WHO is based on literature that builds from the suggestion made by the United States Interdepartmental Committee on Nutrition for National Defence (ICNND) for surveys done among military groups in 1963 [57]. In that survey report, the US ICNND suggested that SR values ranging from 10–20 µg/100 mL (0.35–0.70 µmol/L) indicate low vitamin A whilst those below this range are deficient, and those above it are normal. The rationale for this suggestion was not discussed in this report and the most recent WHO paper that discusses the cut-off value for SR was published in 1996 [7]; nearly three decades ago and still refers to the same cut-off mark. Despite the lack of updating and proper validation of this threshold, it is still used globally to identify VAD in PSC through SR and sometimes RBP concentrations (when it’s used as a proxy), in national and sub-national MNS. This challenge is present with most thresholds of nutrition biomarkers [56,58], especially those where proper development and validation procedures were not followed. After close to 60 years, the cut-off value for SR needs to be reviewed through properly designed studies and updated methods, to determine its accuracy and relevance for different countries and age groups. In light of this, the cut-off value for RBP as a proxy for SR thus needs careful consideration. Further research to better understand the transport mechanisms of SR through RBP, and the concentration differences of Apo- and Holo-RBP under different physiological conditions, could lead to the establishment of better cut-off values for RBP that do not rely on SR.

### 4.4. Policy Implications

The threshold of 0.7 µmol/L gave a prevalence which is higher than the estimated VAD prevalence that was reported using the cut-off value determined through linear regression [23]. The prevalence of 10% appears to be more consistent considering global data [2]. Full coverage of national-level intervention programs is likely required to completely eradicate the problem of VAD [59]. However, in Malawi, this was not the case with uptake of vitamin A capsules in PSC in the six months preceding the MNS of 71% [23]. Furthermore, previous data have shown that most households in Malawi did not meet their vitamin A dietary requirements in the year 2016–2017 [60], that there was, in fact, a decrease in consumption of vitamin A at this point compared to 2010–2011. Considering the likelihood of widespread dietary shortfalls of vitamin A, and the incomplete coverage of supplements, it is likely that some VAD occurs in Malawi. Nonetheless, a VAD prevalence of 10% is below the level considered to be of significant public health concern among PSC [2,61], and this may reflect the success of the vitamin A intervention programmes in the country.

A strength of this study is the examination of inflammation markers in conjunction with vitamin A biomarkers to potentially improve the accuracy of VAD prevalence estimates. However, a major challenge was the apparent bias between SR and RBP concentration data which compromised the analysis. The lack of QC information for the SR analysis made it impossible to identify the source of this bias. Future MNS should require high standards of analytical QC and report the QC data to aid interpretation. If there remain issues in the regression of SR against RBP, then it may be preferable to use the WHO-recommended cut-off SR concentration of 0.7 µmol/L to define deficiency in RBP data.

## 5. Conclusions

RBP is a simple biomarker to work with, but lack of an acceptable cut-off limits its use. The linear relationship of SR and RBP is easily affected by biological and analytical factors and linear regression is too simple a method to account for all these confounders. Furthermore, SR is comparatively challenging to measure and improved analysis and reporting standards are required to ensure accuracy of the reported data. Further research is required to develop robust statistical methods to define cut-off values of RBP concentration indicating VAD, with the aid of well-designed studies. When RBP is used to assess population-level VAD the topic of cut-off values should be approached cautiously to avoid over or underestimating the burden of the problem, which could be risky when this information is adopted by policy advisers and decision-makers. Lastly, vigorous testing of more accurate methods for assessing VAD, such as the SR isotope technique could lead to validation of a more accurate, simple and affordable VAD biomarker.

## Figures and Tables

**Table 1:** Characteristics of vitamin A and inflammatory biomarkers in the sub-sample used for linear regression of pre-school children in Malawi.

	Sub-Sample		Full Sample			
**Biomarkers**	***n***	**mean ± SD**	***n***		**mean ± SD**	***p***
Age (mo)	72	33 ± 14	1100		32 ± 15	0.74
RBP (µmol/L)	72	0.90 ± 0.25	1100		0.89 ± 0.27	0.88
SR (µmol/L)	72	0.98 ± 0.35	1100		-	-
CRP (mg/L) *	72	1.00 (0.26–3.22)	1100		1.47 (0.41–4.7)	0.08
AGP (g/L) *	72	0.95 (0.59–1.43)	1100		1.15 (0.74–1.83)	0.00
**Inflammatory Markers (95% CI)**						
Normal (%)	32	57 (41;72)		450	43 (38;49)	
Total inflamed (%)	40	43 (28;59)		650	57 (52;63)	
Incubation (%)	2	4 (0;14)		14	1 (0;2)	
Early stage (%)	19	18 (10;31)		264	23 (19;28)	
Late stage (%)	19	21 (12;34)		372	33 (29;37)	

Fisher’s exact *t*-test was used for these tests due to the small sample size of the sub-sample. RBP: Retinol Binding Protein, SR: Serum Retinol, CRP: C-reactive protein, AGP: alpha-1-acid glycoprotein. * geometric means were used and are presented after exponentiation, Interquartile ranges are presented in brackets. normal (CRP < 5 mg/L and AGP < 1.0 g/L), incubation (CRP > 5 mg/L and AGP < 1.0 g/L), early convalescence (CRP > 5 mg/L and AGP > 1.0 g/L), and late convalescence (CRP < 5 mg/L and AGP > 1.0 g/L).

**Table 2:** Associations between the vitamin A biomarkers and the inflammatory markers to test for effects of inflammation in the sub-sample.

Biomarkers	*n*	Intercept (95% CI)	Beta Coefficient	
			CRP (95% CI)	AGP (95% CI)
**Bivariate Analysis**				
RBP	72	0.98 (0.91; 1.05)	−0.01(−0.01; 0.00)	NA
		1.09 (0.95; 1.23)	NA	−0.13 (−0.23; −0.03)
SR	72	1.01 (0.94; 1.14)	−0.01(−0.01; 0.00)	NA
		1.04 (0.85; 1.24)	NA	−0.04 (−0.18; 0.10)
**Multivariate Analysis**				
RBP	72	1.05 (0.92; 1.19)	−0.01 (−0.01; 0.00)	−0.06 (−0.17; 0.04)
SR	72	1.00 (0.80; 1.20)	−0.01 (−0.01; 0.00)	0.04 (−0.12; 0.19)

RBP: Retinol Binding Protein, SR: Serum Retinol, CRP: C-reactive protein, AGP: alpha-1-acid glycoprotein.

**Table 3:** Model outputs of the linear regression and correlation coefficients before and after correcting for confounders.

Correction Method	Linear Equation Coefficients		Correlation Estimate	R^2^	Calculated Cut-Off (µmol/L)
	Intercept	Beta			
Original regression (model 1)	0.43	0.62	0.45	0.20	0.45
Categorical inflammation adjusted regression (model 2)	0.35	0.64	0.42	0.18	0.42
Incubation	-	0.11			
Early	-	0.09			
Late	-	0.09			
Continuous inflammation adjusted regression (model 3)	0.37	0.60	0.47	0.22	0.43
CRP	-	0.00			
AGP	-	0.07			
Removing inflamed participants (model 4)	0.47	0.53	0.44	0.20	0.43

All coefficient values in this table are exponentiated, Model 3 had a smaller sample size (n = 32) after removing n = 40 inflamed participants. Inflammation was defined as: normal (CRP < 5 mg/L and AGP < 1.0 g/L), incubation (CRP > 5 mg/L and AGP < 1.0 g/L), early convalescence (CRP > 5 mg/L and AGP > 1.0 g/L), and late convalescence (CRP < 5 mg/L and AGP > 1.0 g/L).

**Table 4:** Point estimates of vitamin A deficiency assessed using retinol-binding protein and inflammation assessed using C-reactive protein and Alpha-1-acid glycoprotein.

**Biomarkers**	***n***	**Prevalence % (95% CI)**		
		**0.43 µmol/L**		**0.7 µmol/L**
Unadjusted RBP	1100	2 (1; 4)		24 (20; 29)
BRINDA adjusted RBP	1100	0 (0; 1)		10 (7; 14)
**Inflammation Criteria**				
	n		% (95% CI)	
Total	1100		57 (52; 63)	
Incubation stage	14		1 (0; 2)	
Early stage	264		23 (19; 28)	
Late stage	372		33 (29; 37)	

RBP: Retinol Binding Protein. Inflammation was defined as: normal (CRP < 5 mg/L and AGP < 1.0 g/L), incubation (CRP > 5 mg/L and AGP < 1.0 g/L), early convalescence (CRP > 5 mg/L and AGP > 1.0 g/L), and late convalescence (CRP < 5 mg/L and AGP > 1.0 g/L).

## Data Availability

Data used in this study is available on the DHS program website and access to it can be granted upon a reasonable request.

## Supplementary Materials

The following are available online at https://www.mdpi.com/2072-6643/13/3/849/s1, Figure S1: Flow chart of study participants, showing the full study sample as well as the study sub-sample.
