# Supplementary material for: Re-Defining the Population-Specific Cut-Off Mark for Vitamin A Deficiency in Pre-School Children of Malawi
Source: Nutrients. 2021 Mar 5;13(3):849. doi: 10.3390/nu13030849 (PMC8000145; doi:10.3390/nu13030849)
Supplement: Supplementary file 1 [file nutrients-13-00849-s001.pdf]

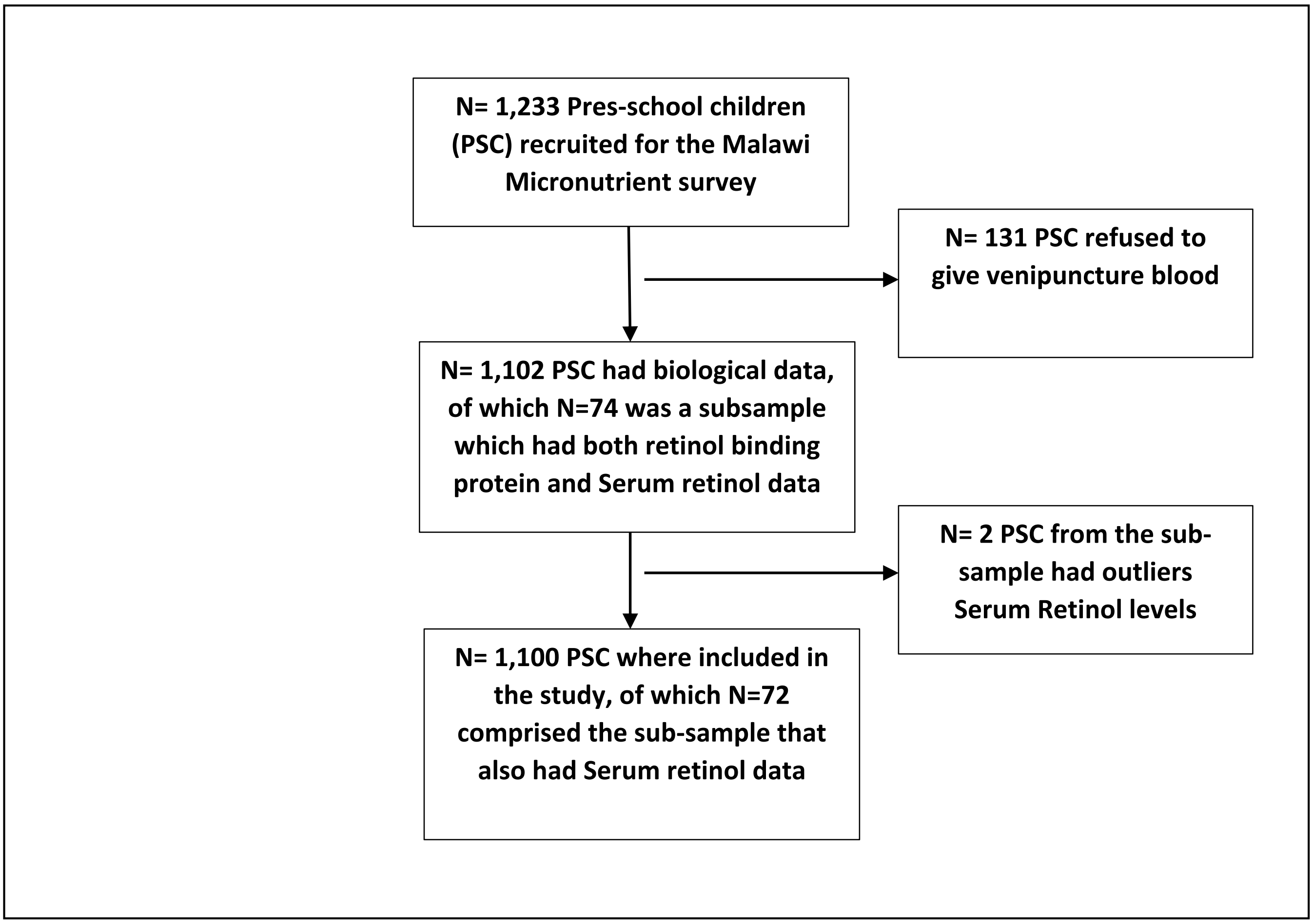

Figure S1. Flow chart of study participants, showing the full study sample as well as the study sub-sample.
